# Supplementary material for: Trend and determinants of home delivery in Gambia, evidence from 2013 and 2020 Gambia Demographic and Health Survey: A multivariate decomposition analysis
Source: PLoS One. 2023 Dec 6;18(12):e0295219. doi: 10.1371/journal.pone.0295219 (PMC10699591; doi:10.1371/journal.pone.0295219)
Supplement: S1 File — (DOCX) [file pone.0295219.s001.docx]

Supplementary file 1. Questionnaire.

| Socio-demographic factor | | | |
| --- | --- | --- | --- |
| S.no | Variable | Categories | DHS code |
| 101 | Cluster number | …………. | V001 |
| 102 | Sampling weight | …………. | V005 |
| 103 | Women age | …………… | V531 |
| 105 | Residence | 1. Urban 2. Rural | V025 |
| 106 | Women highest educational level | 1. No educated 2. Primary education 3. Secondary education 4. Higher | V106 |
| 107 | Residence | 1. Banjul 2. Kanifing 3. Brikama 4. Mansakonko 5. Kerewan 6. Kuntaur 7. Janjanbureh 8. Basse | V024 |
| 108 | Religion | 1.Islam  2. Christianity | V130 |
| 109 | Employed status of women | 1. not working and didn't work in last   2. Managers  3.professionals  4.technicians and associate professionals  5. clerical support workers  6. service and sales workers  7.skilled agricultural, forestry and fish  8. craft and related trades workers  9.plant and machine operators, and assemble  10. Elementary occupations 11. armed forces occupations  12. Others | V716 |
| 110 | Husband highest educational level | 1. No educated  2. Primary education  3. Secondary education  4. Higher | V701 |
| 111 | Wealth index | 1. poorest 2. poorer 3. middle 4. richer 5. richest | V190 |
| 112 | Number of living children (parity) | _________ | V218 |
|  | Number of antenatal visits during pregnancy | ________ | M14_1 |
| 113 | Place of delivery | 1.Respondent's home  2.Other home  3.Government hospital  4.Government health center  5.Government health post  6.Private hospital/clinic  7.NGO hospital/ clinic  8. Other private medical sector | M15_1 |
| 114 | Total children ever born | _______ | V201 |
| 115 | Covered by health  insurance | 1. No 2. Yes | V481 |
| 116 | Getting medical help for self | 1.big problem  2.not a big problem | V467d |
