# Supplementary material for: Trend and determinants of home delivery in Gambia, evidence from 2013 and 2020 Gambia Demographic and Health Survey: A multivariate decomposition analysis
Source: PLoS One. 2023 Dec 6;18(12):e0295219. doi: 10.1371/journal.pone.0295219 (PMC10699591; doi:10.1371/journal.pone.0295219)
Supplement: Attachment — Submitted filename: point_by_point response.docx [file pone.0295219.s002.docx]

Date: September 17, 2022

To:	“PLOS ONE Editorial Office" PONE-D-22-03923

From:	"Solomon Gedlu" sol.gondar@gmail.com

Subject: Author’s response to reviewers’ comments

Trend and determinants of Home Delivery in Gambia, Evidence from 2013-2020 Gambia Demographic and Health Survey: A Multivariate decomposition analysis.

Solomon Gedlu^1^

1Department of Epidemiology and Biostatistics, Institute of Public Health, University of Gondar, Gondar, Ethiopia.

Dear Editor,

We would like to express my appreciation for the pertinent comments and helpful suggestions from the reviewers on my manuscript. Based on the reviewers’ comments and suggestions, I further revised the manuscript and made corrections and modifications accordingly. Below are attached my point-by-point responses to the comments and suggestions. I hope that the revised manuscript address fully the reviewers’ comments. Finally, appreciate your kind suggestion and comments on my work manuscript.

Sincerely yours,

Solomon Gedlu Nigatu, author

**Point-by-point response**

**Response to Reviewer #1**

**Comment 1**: A major revision is needed on the English language and the flow information.

**Author response 1:** Dear reviewer thank you so much for your constructive comment. As you saw and I also checked the write-up had a huge language error. I took a series of measures to correct all language related issues.

**Comment 2**: Discussion and conclusion sections require major revision.

**Author response 2:** Dear reviewer thank you so much. As per your request and I checked not only the mentioned parts but also additional parts of the manuscript that had unclarity. I hope the revised looks good to the reader.

**Comment 3**: The statistical methodology used in the study requires detail and clear explanation with the result/findings.

**Author response 3:** Dear reviewer thank you so much for your valuable comment. Yes, to make the paper more concise I missed some important information which deal about the analysis I used. On the revised manuscript I did my best to explain what does mean decomposition analysis and how the analysis was done.

**Comment 4**: Page 2; Line 26: Gambia Demographic and Health Survey

**Author response 4:** Dear reviewer thank you so much. I corrected it.

**Comment 5**: Page 3; line 42: in this study ….

**Author response 5:** Dear reviewer thank you so much for your suggestion. I made corrections as per the suggestion.

**Comment 6**: Page 6; line 120-121: dependent variable and response variable: using one of the two word is better for easily understanding and uniformity of using the term.

**Author response 6:** Dear reviewer thank you so much. I used these two words interchangeably but to avoid confusion for the readers I used the dependent variable.

**Comment 7**: Page 6; line 124-125: clinics mentioned two times.

**Author response 7:** Dear reviewer thank you so much. I deleted it.

**Comment 8**: Page 6; line 124: nongovernmental hospital: is it different from private hospital. If the two are similar, cancel one of the two.

**Author response 8:** Dear reviewer thank you so much for your curiosity question. There is no difference between the hospitals regarding the delivery service. So, I mentioned only private hospitals.

**Comment 9**: Page 7; line 127-128: move it to ethical considerations section.

**Author response 9:** Dear reviewer thank you so much. I did as per your recommendation.

**Comment 10**: Page 7; line 128-130: move it to the end of “method of data analysis” section.

**Author response 10:** Dear reviewer thank you so much. Your recommendation sounds, so I did it.

**Comment 11**: Page 8; line 151: 63.7%

**Author response 11:** Dear reviewer thank you so much. I missed the percentage. Now it is corrected.

**Comment 12**: Page 8; line 158: study: survey

**Author response 12:** Dear reviewer thank you for understanding me. It was wrong to say study. On the revised it is correct.

**Comment 13**: Page 12: line 247: “ ….. change in the behavior of the study participants …”. How you could proof it?

**Author response 13:** Dear reviewer thank you so much for your curiosity question. In decomposition analysis, if the trend is significant, the contributor variables for the change are either characteristic effect or coefficient effect. Sometimes both will contribute to the change. Keep in mind this, the coefficient effect can be mentioned using different terms like effect, coefficients, and behavioral. In the revised manuscript I used the term coefficient effect consistently.

**Response to Reviewer #2**

**Comment 1:** The subject trend and determinants of home delivery is important and likely contributor factors may differ from region to region. Thus, this might be useful study in assessing the likely predictors in the study area. However, the most important could be to assess the trend and magnitude of home delivery when home delivery was high (36.18% in 2013 GDHS) and factors associated when the home delivery rate has dropped to14.39% (in 2019-2020 GDHS). In the current form, the results do not add to the existing knowledge about the factors that are associated with home delivery. Moreover, the other issue here is a woman can give a birth at home by skilled birth attendants. So it might be very useful to assess the trend and determinants of skilled delivery instead of home delivery versus health facility delivery.

General comments: document lacking grammatical, check for sentence verb agreement, spelling and coherence. I suggest for English edition.

**Author response1:** Dear reviewer thank you so much for your constructive comment. In Gambia maternal mortality ratio is still high. Even though the data set does not clearly show how that specific home birth assisted, birth takes place at home will not be supported by a skilled professional. I believe this manuscript will contribute to the knowledge as well as indicate possible solutions for the problem. For sure there were tremendous grammatical, spelling, and coherence problems. The author did his all capacity for English edition. I hope the revised edition meet he journal requirement for language clarity.

**Comment 2:** In the abstract and Introduction, the reason for why this study was done not convincing.

**Author response 2:** Dear reviewer thank you so much for your valuable comment. In the revised version more detail regarding the rationale of the study is stated. Please have a look.

**Comment 3:** Abstract: include the odds ratios and CIs in your result section.

**Author response3:** Dear reviewer thank you so much for your suggestion. In a decomposition analysis odds ratio cannot do anything rather the confidence interval with 95% is the most important.

**Comment 4:** Line 34, what the number [34.78, 37.58] stand for? Please use statistical language to report the findings in the result section.

**Author response 4:** Dear reviewer thank you so much for your comment. Sorry for the unclarity happened. It is a confidence interval but it missed CI and 95 %. It is corrected accordingly.

**Comment 5:** The introduction is not clear and difficult to follow. For the example starting from line 82, not clear what is meant “Previous research has uncovered a number of factors that contribute to the decline of home delivery” and the author mentioned some references. And starting from line 86, it stated that “Gambia has a high total fertility rate. Therefore, women gave birth to an average of 4.4 children.......” The author should rewrite the introduction so that it will be easier to follow.

**Author response 5:** Dear reviewer thank you so much for your comment. The paragraph has a grammatical error. So, it was rewritten to give a clear message.

**Comment 6:** Line 108, First mention the source of data. Perhaps you can change the subsection “ Study population and data collection” to Data sources and study population. First mention what is your data sources before describing the data collection produces they have used. Moreover, the dependent and independent variables should not be in the study population and data collection. They should be in a separate sub section: study variables. Line 122, the author mention that the response variable is a binary outcome, with 1 indicating that the womon gave birth at home or in another home. Do you mean at her home or another home. Please correct this. The dependent and independent variables should be in a separate subsection “study variables”. And rewrite the sentences for the dependent variable. Include the independent variables and state the variables in detail.

**Author response 6:** Dear reviewer thank you so much for your constructive comments. It is stated as "Data source, Study population, and sampling technique” in the revised manuscript. I also put the study variable in a sub section as dependent and independent variables and encompassed all the details. The phrase “woman gave birth at home or in another home” I wanted to say in her home and correction is done on it.

**Comment 7:** In the study area, it is important to describe the study area in more detail. For example the estimated populations living in rural and urban areas, and the number of health facilities in the region.

**Author response 7:** Dear reviewer thank you so much for your constructive comments. Yes, the most important information was missed. In the revised manuscript all the requested information is included.

**Comment 8:** The method section is difficult to follow. The author should rewrite the method section so that it will be easiy to follow. For example method for data analysis the author should focose on the techniques that used to address the objective, instead of discussing the meaning of confidence intervals.

**Author response 8:** Dear reviewer thank you so much for your constructive comments. I also checked it and it needs much improvement. So, I did a lot of effort to make it easily understandable to the readers.

**Comment 9:**The result section is difficult to follow. Please consider rewording the paragraphs to make it easier to follow.

**Author response 9:** Dear reviewer thank you so much for your constructive comments. The same is true for this section like the method part. I hope now it sounds.

**Comment 10:** Line 151, change the value 97% to 98%.

**Author response 10:** Dear reviewer thank you so much. It should be 97.9%. Now the error is fixed.

**Comment 11:** Lines 158-160: Lack clarity, not clear what is meant by “Home delivery was 36.18 % [34.78, 37.58] in Gambia in the first study. After five years, another survey was conducted in 2019-2020...”

**Author response 11:** Dear reviewer thank you so much for your constructive comments. The author wanted to mention the point estimation of home delivery with its respective confidence interval.

**Comment 12:** Grammatical errors are present sporadically across the write-up. English needs to be corrected.

**Author response 12:** Dear reviewer thank you so much for your constructive comment. Yes, indeed there were several grammatic errors as explained previously, I did all effort to overcome such a problem.

**Comment 13:** If the data is reanalyzed in light of the above suggestion, the discussion need to be aligned with the revised analysis and results. The findings should be sufficiently discussed in comparison with previous research studies and the existing differences should be fully clarified. For example, what is the new finding which differ from existing literatures. Currently the discussion does not discuss in depth to add to the existing knowledge about factors that determine access to health faculties delivery. Discussion needs to be strengthened..

**Author response 13:** Dear reviewer thank you so much for your constructive comment. I do not think there is a need to have a reanalysis. I strongly believed the analysis was well done but the way I write was not good enough. So, the revised manuscript considered all the comments including language. If there is a need to improve, I am open to doing so.
