# Supplementary material for: Trend and determinants of home delivery in Gambia, evidence from 2013 and 2020 Gambia Demographic and Health Survey: A multivariate decomposition analysis
Source: PLoS One. 2023 Dec 6;18(12):e0295219. doi: 10.1371/journal.pone.0295219 (PMC10699591; doi:10.1371/journal.pone.0295219)
Supplement: Attachment — Submitted filename: Trend and determinants of Home Delivery in Gambia.docx [file pone.0295219.s003.docx]

**Trend and determinants of Home Delivery** **in Gambia, Evidence from 2013-2020**

**Gambia Demographic and Health Survey: A Multivariate decomposition analysis**

The author did a good job in this document but the document is far from acceptable academic writing.

Abstract

- At the onset, home delivery should be clearly and explicitly defined, which is not the case. I suggest something like defining the main variable, describing its severity, making a connection with Gambia, and the gap, and showing how your objective will solve the gap.
- The current introduction does not make any sense in an academic writing context and should be rewritten.
- Look at the first two sentences, they are just connected and are not clear
- Overall, the abstract is good but confusing and does not leave any impressive impression. I suggest redrafting it according to the following guidelines. The first two lines must encompass the context of the study and the research problem, further two lines must be covered the objective of the papers with unfolding the description of the title. In the next 2 to 4 lines the methodology will be covered. Afterward, the next two lines are for result and performance. In these lines, the author must define how the results and performance are being achieved, for instance, by conducting either simulation or physical implementation. Please mention the name of the simulation or the physical method. The result statistics must be mentioned in the last two lines and either in percentage or with real-time values.

Introduction

- The authors must give a clear procedure for the proposed solution with an algorithm (via flowcharts or pseudo codes, i.e. the flowcharts of a proposal work must be drawn), and must be supported with a figure either a block diagram of the proposed methodology or the topology in a formal style.
- In the Literature Review section, the authors must cover the shortcomings because shortcoming or challenges in previous work is not mentioned. If the previous work is free of challenges and there is no issue then what motivated the authors to propose this study? It inculcates that authors should revise the literature review and critically highlight the problems in the previous study and compare the proposed solution and tells how the proposed solution is best fitted.
- And please, give special attention to writing English. There are many switching tenses, punctuation errors, poor word choices, and unclear and complicated wordy sentences throughout the document.
- You may add a table and present the summary of related work, the shortcomings, and the proposed solution accordingly.

Methods

- In the Research Methodology section, what is the difference between your proposed method and other based techniques? (II) Please compare your proposed method with other based methods in terms of time complexity.

Results

- Critical analysis of the finding, which is the most important part, is missing. This would help the readers to further improve the study.
- Again, go for a thorough proofread of the paper to rectify several existing typos and grammatical mistakes to improve the written quality of the paper. If necessary, take the help of a native English speaker to improve the language of the paper.

Conclusion

- Regarding the conclusion paragraph, Please precisely describe the outcome of the study and justify the statements that are mentioned in the abstract. Further, it must contain additional points and must give a clear and more discussion about the experimental results. The main novelty and contribution of needs must be summarized and highlight the recommendations based on obtained results. These results are the hallmark for future extension therefore, please spend some more time writing the conclusion and based on the results suggest new directions.

References

While reviewing the references, I observed that cited references are outdated whereas more work already has been done in the proposed study. The cited references are neither sufficient nor suitable and therefore must extend the list and focus only on the papers from the recent 3 years. The following references may be added to supersede the outdated ones for the authors' convenience.
