# Supplementary material for: Trend and determinants of home delivery in Gambia, evidence from 2013 and 2020 Gambia Demographic and Health Survey: A multivariate decomposition analysis
Source: PLoS One. 2023 Dec 6;18(12):e0295219. doi: 10.1371/journal.pone.0295219 (PMC10699591; doi:10.1371/journal.pone.0295219)
Supplement: Attachment — Submitted filename: point_by_point_response.docx [file pone.0295219.s004.docx]

Date: September 9, 2023

To: PLOS ONE Editorial Office. manuscript ID: PONE-D-22-03923

From:	"Solomon Gedlu" sol.gondar@gmail.com

Subject: Author’s response to reviewers’ comments

Trend and determinants of Home Delivery in Gambia, Evidence from 2013 and 2020 Gambia Demographic and Health Survey: A Multivariate Decomposition Analysis.

Solomon Gedlu1

^1^Department of Epidemiology and Biostatistics, Institute of Public Health, University of Gondar, Gondar, Ethiopia.

Dear Editor,

I would like to express my appreciation for all who participant in the review process of my manuscript. Based on the reviewers’ comments and suggestions, I revised it again and made corrections accordingly. I hope that the revised manuscript address fully the reviewers’ comments. I am always willing to improve the manuscript, so please do not hesitate to forward your feedback if there is anything I missed.

Best regards,

Solomon Gedlu Nigatu, author

**Point-by-point response**

Response to Reviewer #3

**Comment:** At the onset, home delivery should be clearly and explicitly defined, which is not the case. I suggest something like defining the main variable, describing its severity, making a connection with Gambia, and the gap, and showing how your objective will solve the gap.

**Author response:** Dear reviewer thank you for your valuable comment. As per the comment and suggestion I incorporate the definition of what does mean home delivery and its magnitude on different scale. All the change is available on revised manuscript.

**Comment 1**: -The current introduction does not make any sense in an academic writing context and should be rewritten. Look at the first two sentences, they are just connected and are not clear. Overall, the abstract is good but confusing and does not leave any impressive impression. I suggest redrafting it according to the following guidelines. The first two lines must encompass the context of the study and the research problem, further two lines must be covered the objective of the papers with unfolding the description of the title. In the next 2 to 4 lines the methodology will be covered. Afterward, the next two lines are for result and performance. In these lines, the author must define how the results and performance are being achieved, for instance, by conducting either simulation or physical implementation. Please mention the name of the simulation or the physical method. The result statistics must be mentioned in the last two lines and either in percentage or with real-time values.

**Author response:** Thank you dear reviewer for your constructive comment. As per the recommendation I made a change on the introduction part of the manuscript. However, Point raised to include simulation or physical implementation is not possible for such kind study. As to my best this is a epidemiological cross sectional study rather than implantation study.

**Comment 1**: -The authors must give a clear procedure for the proposed solution with an algorithm (via flowcharts or pseudo codes, i.e. the flowcharts of a proposal work must be drawn), and must be supported with a figure either a block diagram of the proposed methodology or the topology in a formal style.

**Author response:** Dear reviewer thank you for your valuable comment. This is a health science study to figure what the extent of the public health problem and to know the contributor factors. What dear reviewer recommend like flowcharts or pseudo code may suitable for computer science studies.

**Comment 1**: - In the Literature Review section, the authors must cover the shortcomings because shortcoming or challenges in previous work is not mentioned. If the previous work is free of challenges and there is no issue then what motivated the authors to propose this study? It inculcates that authors should revise the literature review and critically highlight the problems in the previous study and compare the proposed solution and tells how the proposed solution is best fitted.

**Author response:** Thank you dear reviewer for your constructive comment. As far as I know there is no literature review section, so its difficult for me to address what you mention.

**Comment 1**: And please, give special attention to writing English. There are many switching tenses, punctuation errors, poor word choices, and unclear and complicated wordy sentences throughout the document.

**Author response:** Dear reviewer thank you for your valuable comment. You are right there were a number of grammar and punctuation errors. My colleagues and I have strived to avoid the mistakes.

**Comment 1**: You may add a table and present the summary of related work, the shortcomings, and the proposed solution accordingly.

**Author response:** Dear reviewer thank you for your valuable comment. This is basic research for the seek of advancing knowledge on human health issue. For applied (operation) research there a solution and you test the effectives of the proposed solution. For my case it does not work.

**Comment 1**: -In the Research Methodology section, what is the difference between your proposed method and other based techniques? (II) Please compare your proposed method with other based methods in terms of time complexity.

**Author response:** Dear reviewer thank you for your valuable comment. Please view my previous response.

**Comment 1**: Critical analysis of the finding, which is the most important part, is missing. This would help the readers to further improve the study.

**Author response:** Dear reviewer thank you for your valuable comment. For this study I used a decomposition analysis and it presented what does it mean, the formula and how it will interpret in the method part. If there is any thing miss, I am willing to address it again.

**Comment 1**: Again, go for a thorough proofread of the paper to rectify several existing typos and grammatical mistakes to improve the written quality of the paper. If necessary, take the help of a native English speaker to improve the language of the paper.

**Author response:** Thank you dear reviewer for your constructive comment. It addressed well with the help of my colleagues

**Comment 1:** Regarding the conclusion paragraph, please precisely describe the outcome of the study and justify the statements that are mentioned in the abstract. Further, it must contain additional points and must give a clear and more discussion about the experimental results. The main novelty and contribution of needs must be summarized and highlight the recommendations based on obtained results. These results are the hallmark for future extension therefore, please spend some more time writing the conclusion and based on the results suggest new directions.

**Author response:** Dear reviewer thank you for your valuable comment. The general objective of the study was to assess the trend of home delivery and identify factors. As far as I concern the outcome of the study will be what is the trend of home delivery “steeply declined” and I mentioned factors contributed for such decline.

**Comment 1**: While reviewing the references, I observed that cited references are outdated whereas more work already has been done in the proposed study. The cited references are neither sufficient nor suitable and therefore must extend the list and focus only on the papers from the recent 3 years. The following references may be added to supersede the outdated ones for the authors' convenience.

**Author response.** Dear reviewer thanks you for your valuable comment. I tried to incorporate those articles published in last five years. It is hard to get articles published within three years.
